# Supplementary material for: Structure of a full-length bacterial polysaccharide co-polymerase
Source: Nat Commun. 2021 Jan 14;12:369. doi: 10.1038/s41467-020-20579-1 (PMC7809406; doi:10.1038/s41467-020-20579-1)
Supplement: Supplementary file 1 — Supplementary Information [file 41467_2020_20579_MOESM1_ESM.pdf]

Supplementary Information

Structure of a full-length bacterial polysaccharide co-polymerase

Benjamin Wiseman[1*], Ram Gopal Nitharwal[1,2], Göran Widmalm[3] and Martin Högbom[1*]

[1]Department of Biochemistry and Biophysics, Stockholm University, Stockholm, Sweden.
[2]Present address: Department of Biotechnology, School of Interdisciplinary and Applied Sciences, Central University of Haryana, Mahendragarh, Haryana, India.
[3]Department of Organic Chemistry, Stockholm University, Stockholm, Sweden.

* Correspondence: BW, bwise@dbb.su.se, MH, hogbom@dbb.su.se

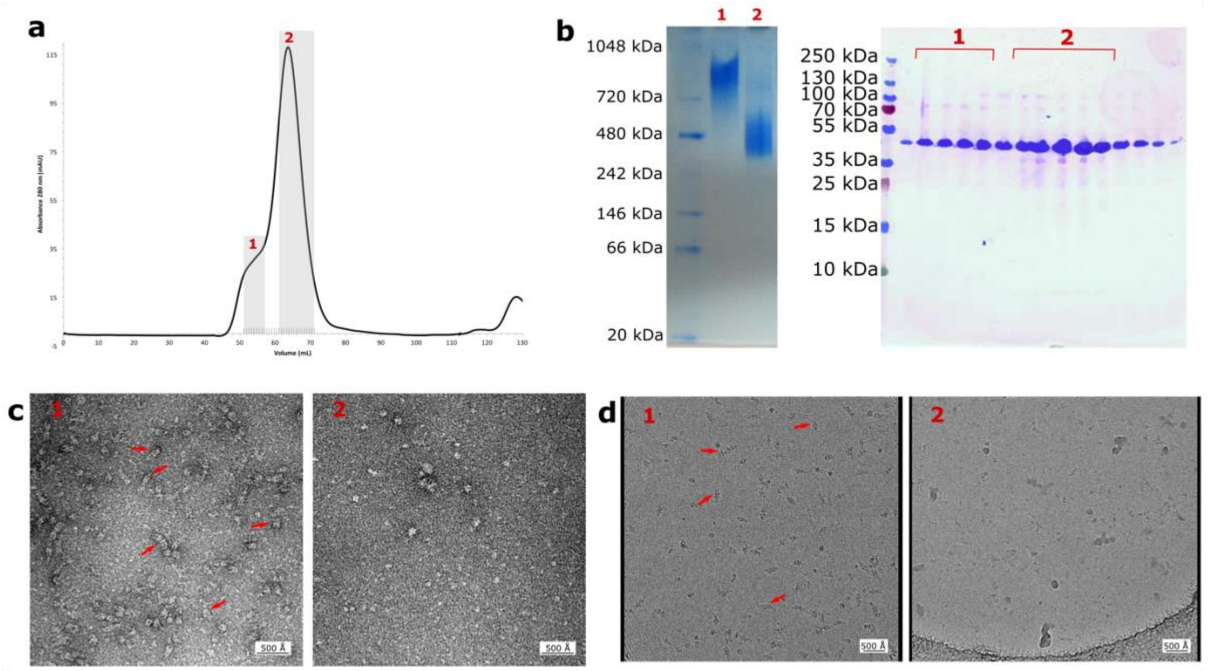

Supplementary Fig. 1 | Purification of WzzB. a, Size-exclusion chromatograph labelled with the two pooled populations. Grey boxes represent the pooled fractions. **b,** Blue-native, and SDS-PAGE respectively of the two populations. **c,** Example negative-stained and **(d)** cryo-electron micrograph of each population. Only population 1 contained usable particles for structural analysis of WzzB (red arrows). Multiple purifications of WzzB were performed resulting in similar size-exclusion chromatographic profiles. From these, a minimum of two independent purifications were visualized using Blue-native PAGE, SDS-PAGE, negative staining, and cryo-electron microscopy with similar results.

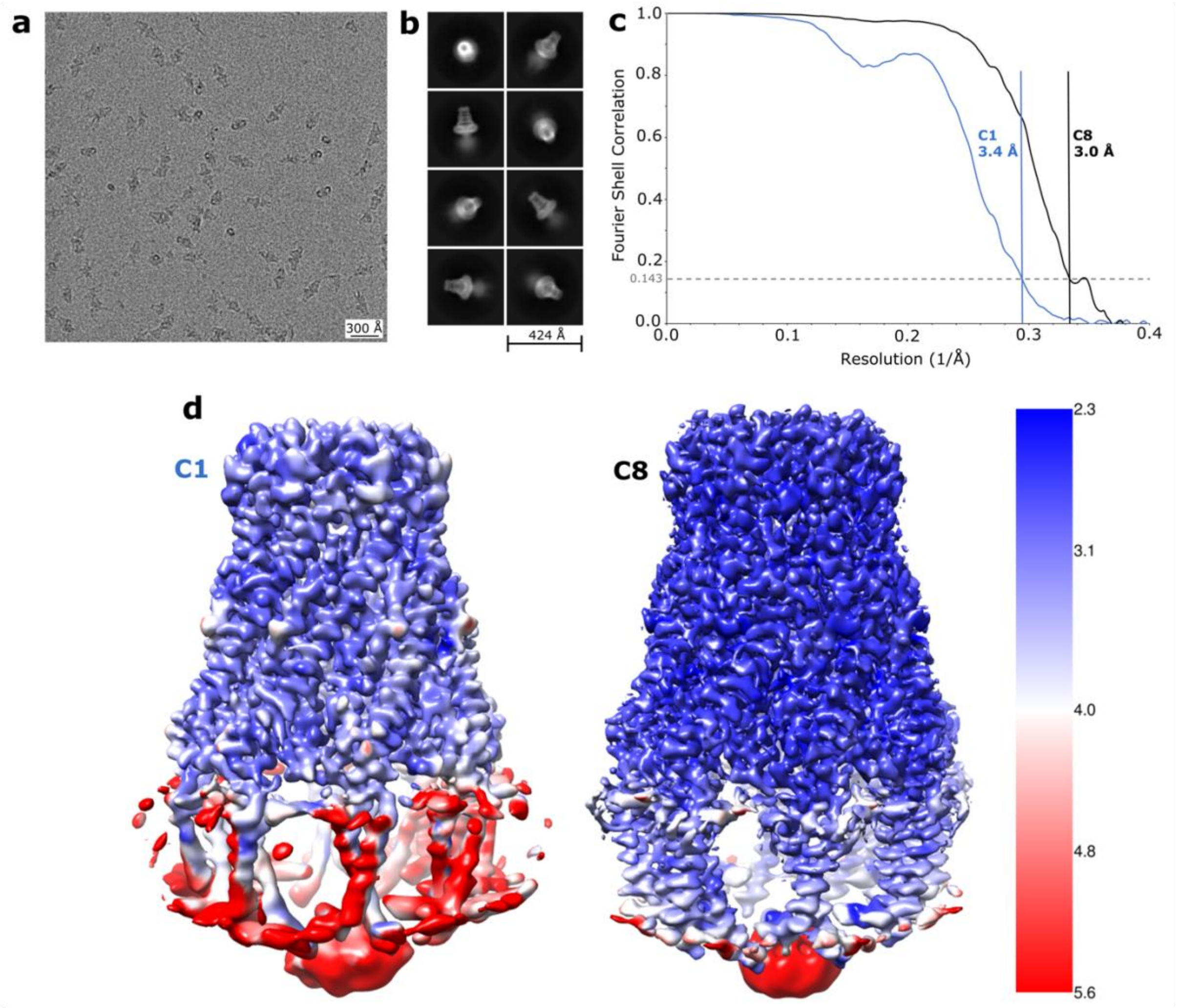

Supplementary Fig. 2 | Cryo-EM data assessment of full-length WzzB. a, Typical micrograph from a single data collection of 2347 micrographs used for automatic particle picking for the 2D classification. **b,** 2D class averages of the WzzB octamer. **c,** Fourier Shell Correlation (FSC) of the final volumes. **d,** Local-resolution estimation of the C1 and C8 density maps.

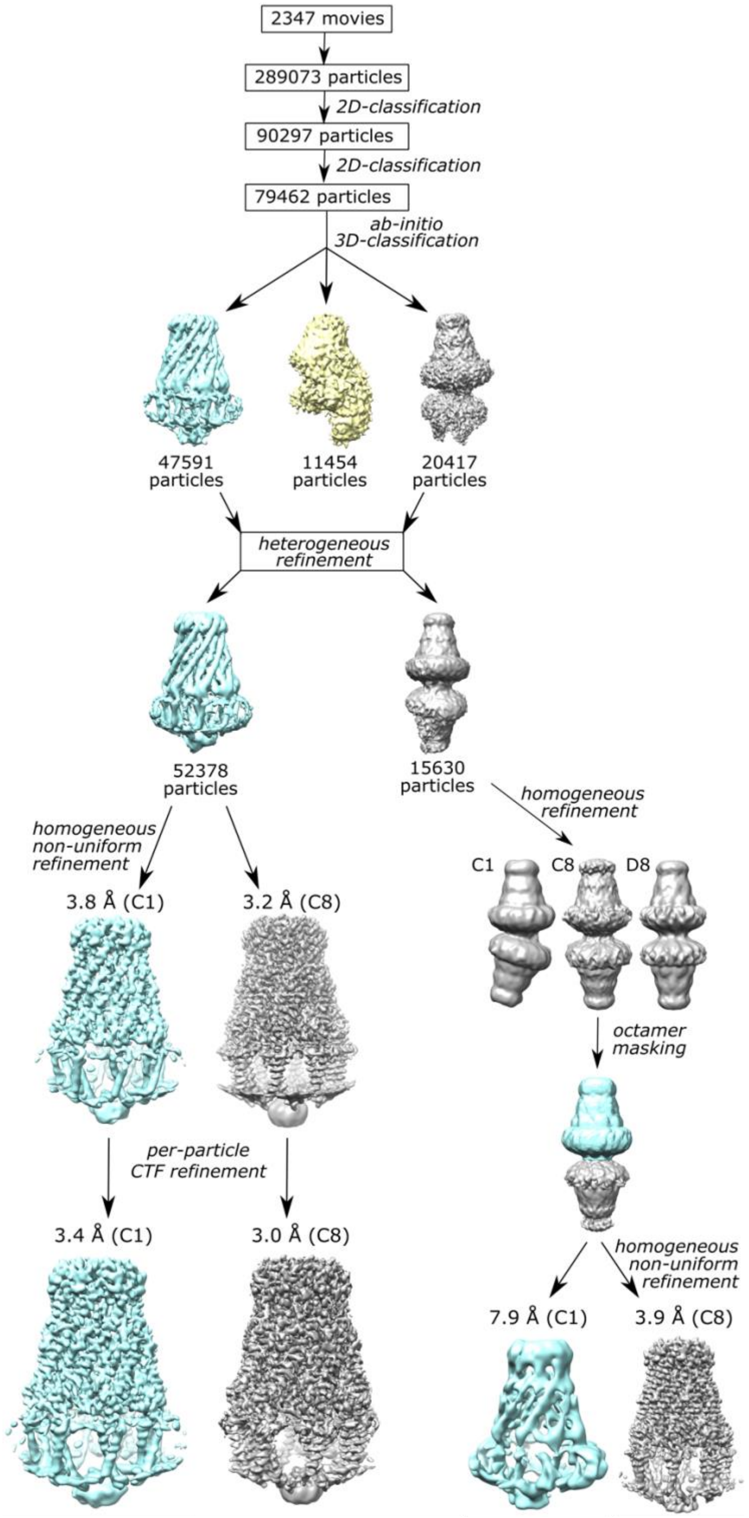

Supplementary Fig. 3 | Cryo-EM particle processing workflow of WzzB. WzzB particles were separated into two distinct species; a single octamer (left) and a dimer of octamers (right). Refinements of the dimers with various symmetries applied resulted in resolutions of 8-10 Å. Masked 3D refinements around a single octamer within the dimeric form resulted in an octameric complex with the same structure as that of the single octamer.

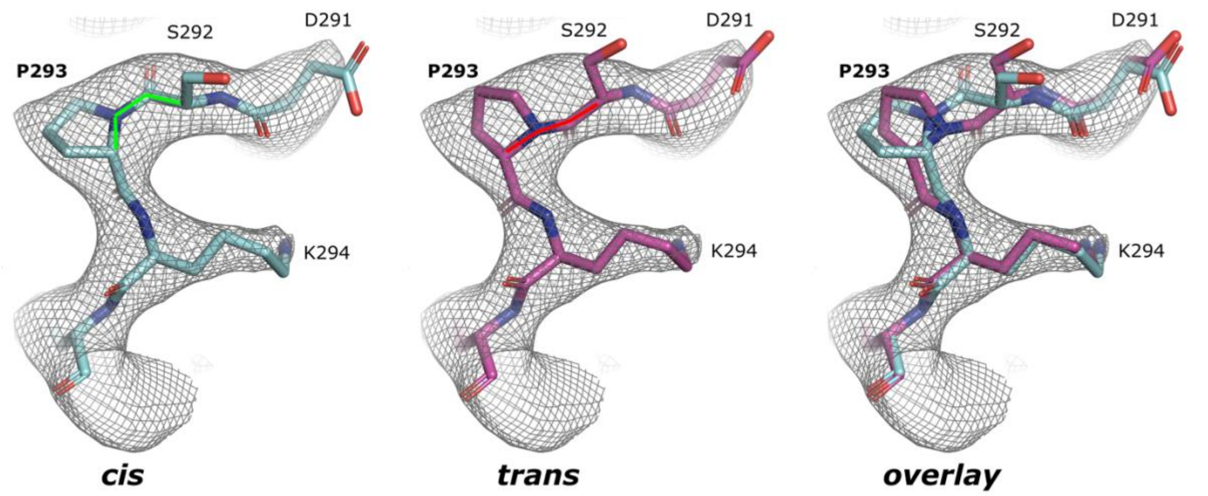

Supplementary Fig. 4 | Comparison of the map-to-model fit of the conserved P293 when in the *cis* and *trans* conformation. The *cis* and *trans* conformation of the ω torsion angle of proline 293 are highlighted by a green and red lines respectively between atoms C_{α}^{S292}-$C(O)^{S292}$-N^{P293}-C_{α}^{P293}.

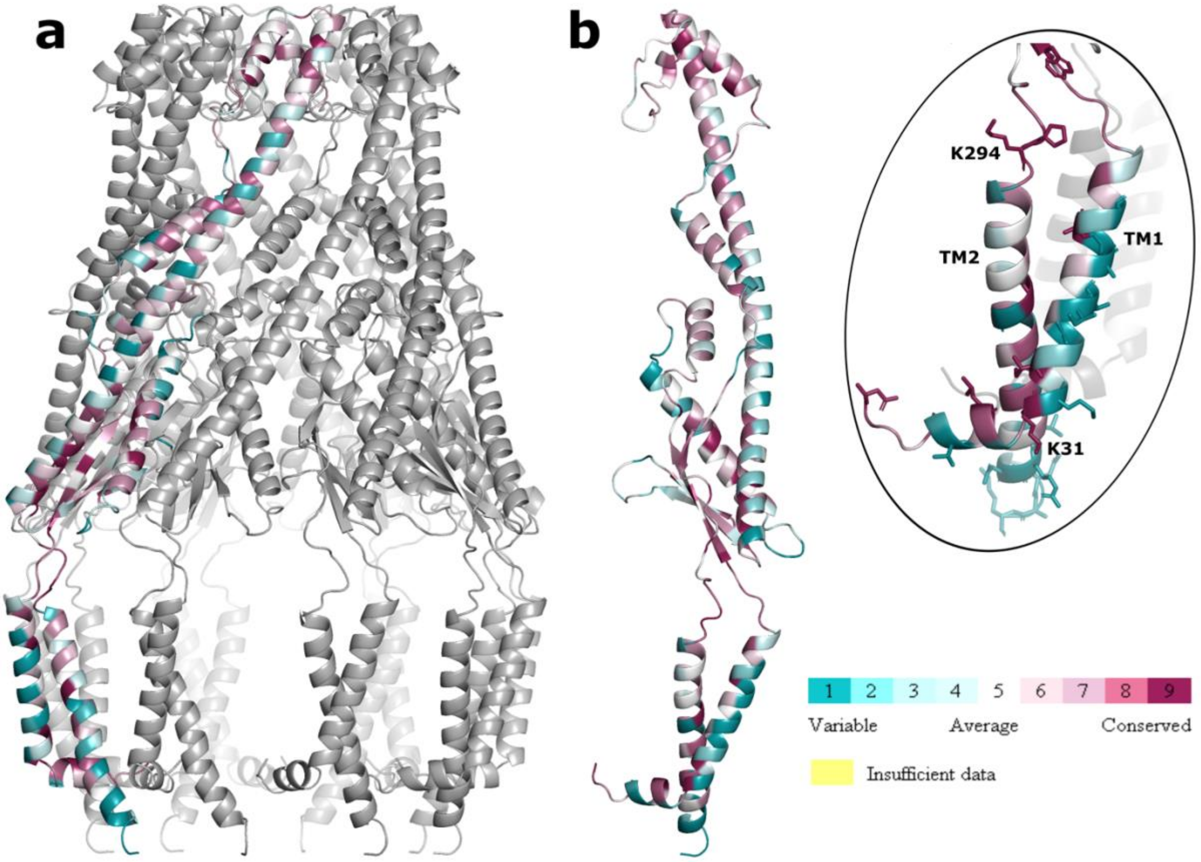

Supplementary Fig. 5 | ConSurf analysis of WzzB. a, The octameric arrangement of WzzB with 1 protomer and **(b),** a single protomer of WzzB colored by sequence conservation as calculated by ConSurf. Circled: zoom of the transmembrane region. The analysis of conserved residues was based on 250 randomly selected members of the polysaccharide co-polymerase family. Residues interacting within a single protomer or with a neighboring protomer within the octamer display a high degree of conservation whereas surface residues are more variable.

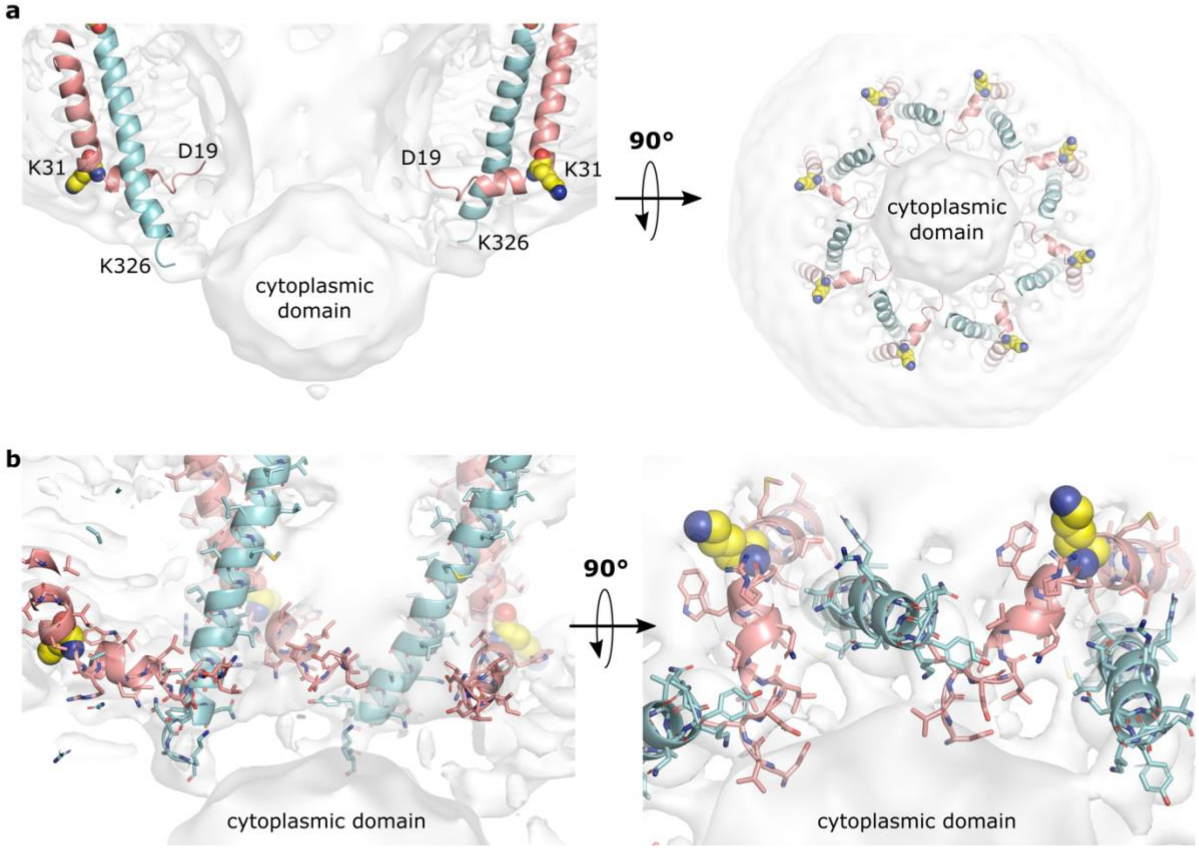

Supplementary Fig. 6 | The area surrounding the cytoplasmic domain. a, Left, sliced to display two opposing protomers showing both the C-terminus of TM2 and the N-terminus of α0 in close proximity to the cytoplasmic domain. Right, view from the cytoplasm. **b,** Zoom to show the interaction of α0 with adjacent TM2s. Right, view from the cytoplasm. The C8 symmetrized WzzB map is displayed in white.

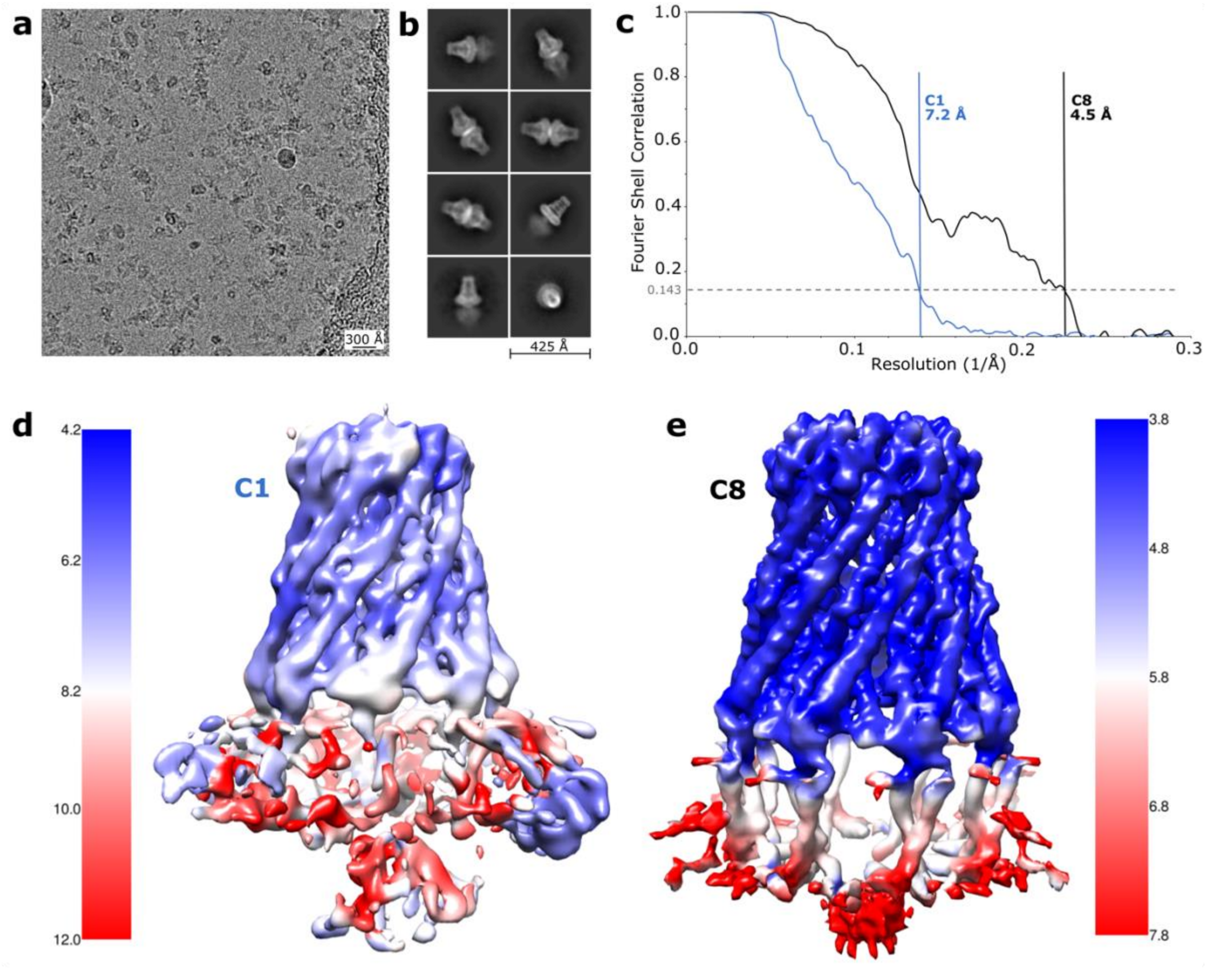

Supplementary Fig. 7 | Cryo-EM data assessment of N-terminally truncated WzzB. Typical micrograph from a single data collection of 2050 micrographs used for automatic particle picking for the 2D classification. **b,** 2D class averages of the N-terminally truncated WzzB. **c,** Fourier Shell Correlation (FSC) of the final volumes. **d,** Local-resolution estimation of the C1 and **(e)** C8 density maps. Since N-terminally truncated WzzB contained only particles of the dimeric form, the maps are a result of masked 3D refinements around a single octamer within the dimeric form.

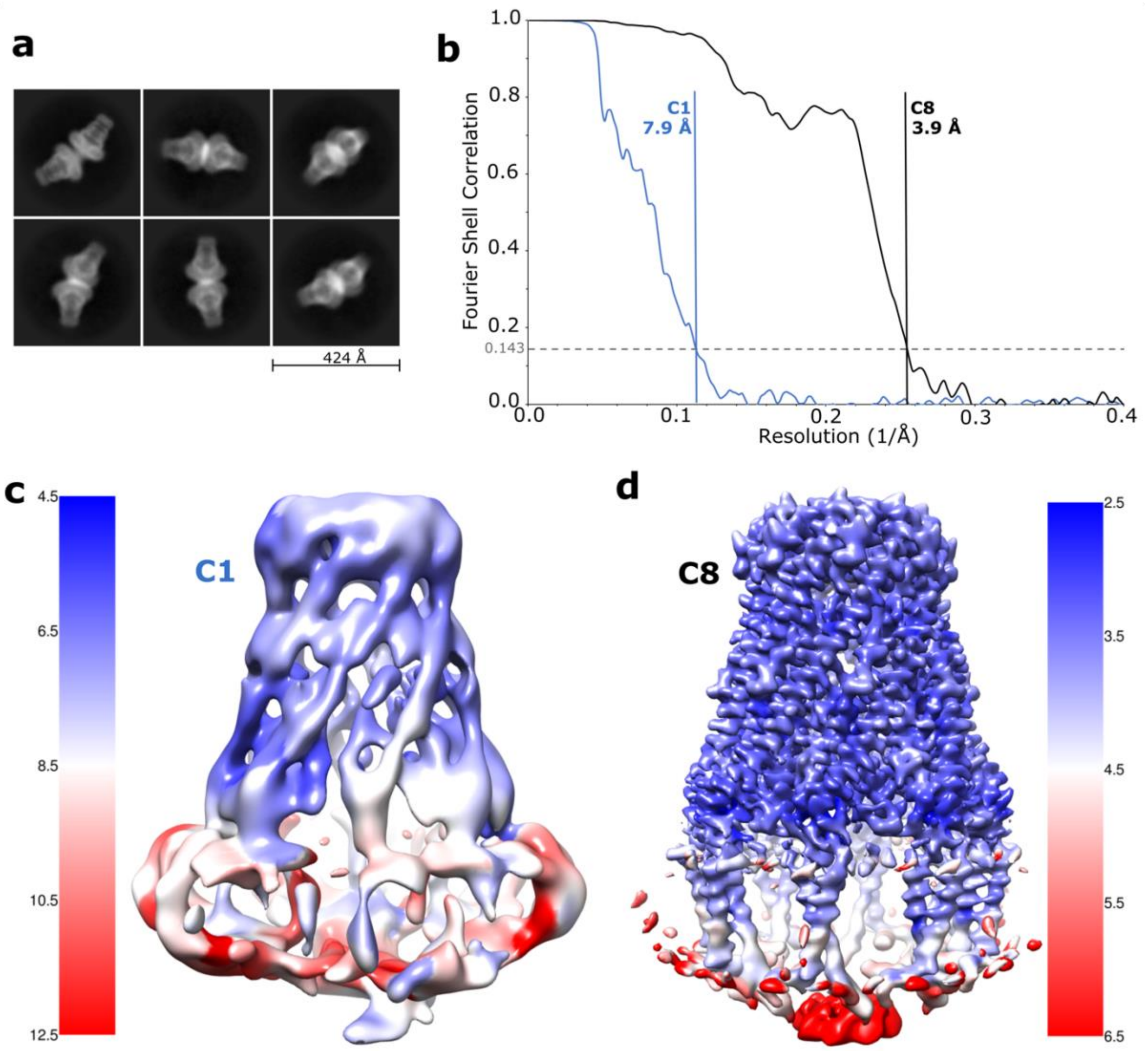

Supplementary Fig. 8 | Cryo-EM data assessment of a single WzzB octamer within the dimeric form. a, 2D class averages of the dimeric form of WzzB. **b,** Fourier Shell Correlation (FSC) of the final volumes. **c,** Local-resolution estimation of the C1 and **(d)** C8 density maps. The maps are a result of masked 3D refinements around a single octamer within the dimeric form.

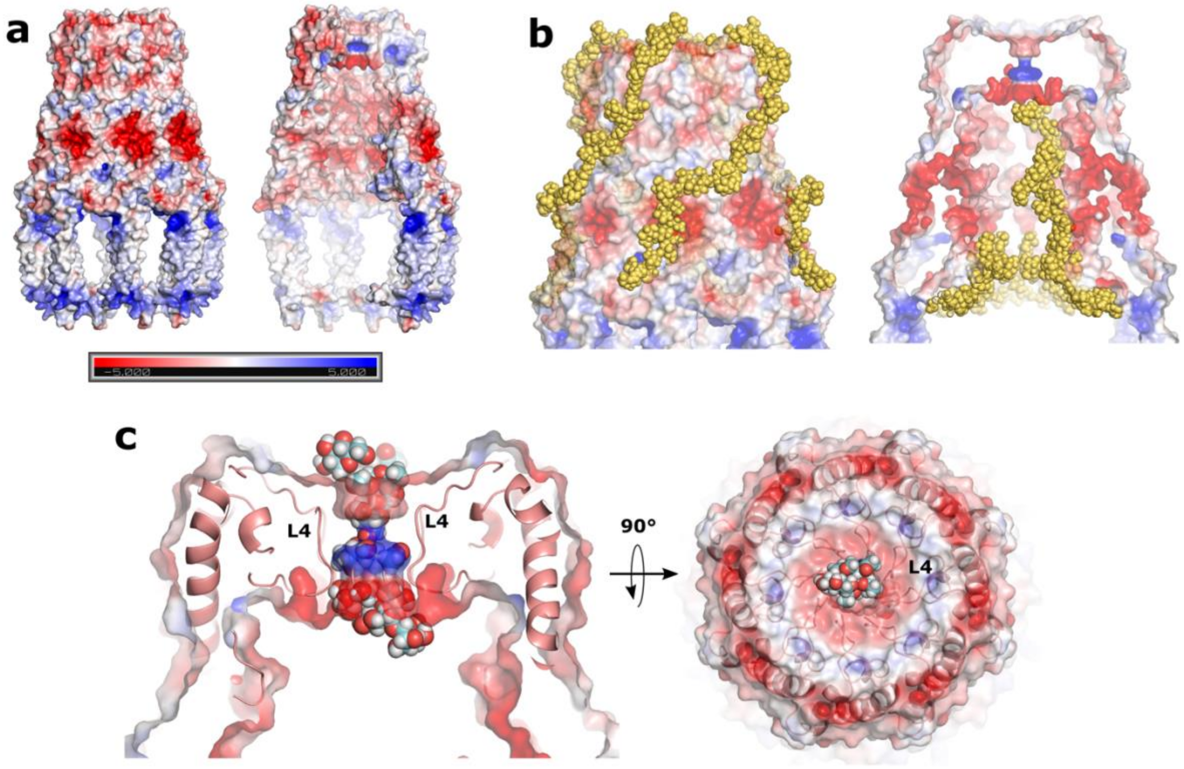

Supplementary Fig. 9 | Schematic of potential association of O16 polysaccharides along the surface of WzzB. a, Electrostatic surface potential of WzzB. Left, the full octameric complex. Right, with 3 protomers removed to display the interior. **b,** Hypothetical model of polysaccharide interaction with WzzB. Left, four copies of an 8 RU *E. coli* O16 O-antigen polysaccharide manually docked to the exterior WzzB. Right, eight copies of a 3 RU and a single 6 RU *E. coli* O16 O-antigen polysaccharide manually docked to the interior of WzzB sliced to display the interior of the WzzB. The polysaccharide was built using LPS Modeler and then manually docked using UCSF Chimera. The surface representation of WzzB is colored by electrostatic surface potential. **c,** Electrostatic surface potential of WzzB overlaid with a representative blindly docked model of a 2-RU O-polysaccharide (shown as spheres) docked to the L4 region of WzzB sliced to display the interaction. Blind docking was performed with SwissDock using a truncated octameric WzzB (residues 183-255, salmon cartoon) and a polysaccharide comprised of 2 RUs of the O16-antigen. The molecules were prepared for docking by adding hydrogens and charges using the Dock Prep feature in UCSF Chimera. The same scale of electrostatic potential was used for all panels and was calculated using the APBS plugin within Pymol.

Table S1. Cloning primers for full-length and N-terminally truncated *E.coli* K12 *wzzB*.

Primer Name	Sequence (5' → 3')	Restriction site
EcWzzB_fwd	ACTCAGCTCGAGATGAGAGTAGAAAATAATAATGTTTCTGG	*XhoI*
EcWzzB_15_fwd	ACTCAGCTCGAGATGCAGATTGATTTGATTGATTTACTAGTGC	*XhoI*
EcWzzB_rev	ATCGACGAATTCCTTCGCGTTGTAATTGCG	*EcoRI*
